# Micro-CT analysis reveals porosity driven growth banding in Caribbean coral *Siderastrea siderea*

**DOI:** 10.1038/s41598-025-90125-w

**Published:** 2025-02-19

**Authors:** James Vincent, Tom Sheldrake

**Affiliations:** Department of Earth Sciences, University of Geneva, Genève, Switzerland

**Keywords:** Scleractinian, Tomography, Segmentation, Caribbean, Seasonality, Density banding, Palaeoclimate, Marine biology

## Abstract

X-radiography of massive scleractinian coral skeletons reveal light and dark couplets termed “growth bands”, which are commonly related to seasonal fluctuations in environmental parameters including insolation and sea surface temperature (SST). Massive corals grow by extension of skeletal structures followed by thickening within the surface tissue layer. Therefore, an understanding of the depth in which skeletal thickening occurs is important to aid the interpretation of seasonal banding patterns. In this study, two colonies of Caribbean coral *Siderastrea siderea* were sampled from the north-west coast of Barbados at water depths of 5 and 15 m. The three-dimensional skeletal structure of each sample was reconstructed at high spatial resolutions using micro-computed tomography (µCT) scanning. A pixel segmentation algorithm was developed to classify different microstructures within the skeleton and to quantify spatial variations in corallite and theca porosity at the micrometer scale. The porosity reconstructions of the deeper sample reveal clearer growth banding, with a more dominant signal originating from within the corallite. Skeletal thickening occurs within the top two-thirds of the total depth of soft tissues and the rate of thickening varies between microstructures. Seasonality in the shallower sample is less clear, although porosity variability with depth is more similar across microstructures. The difference in signal origin and clarity between the two samples is attributed to the varying stability of water depth-dependent variables (i.e., insolation and wave energy). This study provides a new, powerful method of reconstructing and understanding growth strategies in massive scleractinian corals.

## Introduction

Skeletal growth banding in massive scleractinian corals evidences the close and complex relationship between corals and their surrounding environment^1–5^. Recognised as one of the most important archives for paleoclimate reconstructions in the tropics, coral skeletons unveil a chronological record of growth patterns related to environmental change^6^ and coral health^7^. X-radiographs perpendicular to the growth axis reveals couplets of low-density bands (LDB) and high-density bands (HDB), in response to seasonal fluctuations in environmental parameters^8,9^. While the process of coral growth banding has been extensively investigated, there remains uncertainty regarding the timing and factors controlling the formation of these bands^10^. Certain studies suggest that HDB are formed during periods of relatively faster calcification associated with warmer SSTs and heightened light intensity, typically occurring in summer months^11–15^. Conversely, other studies observed that HDB are deposited during times of lower SST and reduced light intensity^16–18^.

A proposed reason for this difference in actual and apparent timing of growth banding is the overlap of soft tissues between the newly formed and existing skeleton^19,20^. Skeletal thickening is generally accepted to occur throughout the entire depth of the tissue layer^21^. Consequently, both newly and pre-existing skeleton occupied by soft tissues undergo thickening simultaneously leading to an overlap between the apparent and actual timing of the band formation. Coral tissue thickness (CTT) is observed to vary ten times more than skeletal extension rate^22^ and between different sexes^16,23^.

How corals invest calcification resources into constructing their skeletons will also influence the interpretation of when growth bands form and the significance of the CTT^24^. For example, different growth strategies were observed between two massive scleractinian corals *Porites* and *Orbicella*^25^*.* Extension rates increased as density decreased with increasing SST (i.e., calcification rates) for *Porites*, whilst the opposite was observed for *Orbicella*. The reason for this difference is that the porous skeleton formed in *Porites* allowed soft tissues to penetrate and calcify the preexisting skeleton. Therefore, the apparent timing of HDB or LDB formation depends on the thickness of soft tissues and the linear extension rate^21^. On the other hand, *Orbicella* is solid and does not allow soft tissues to penetrate the skeleton and consequently forms HDB directly^24^.

Understanding the depth of skeletal thickening within soft tissues is critical as many coral-based paleoclimate studies rely on banding chronology for backdating and synchronising geochemical records^26,27^. Poor comprehension of growth banding could cause invalid interpretations of the climate and environmental proxies contained within their skeleton. This study aims to help resolve inconsistencies and explain variability in the growth banding pattern by investigating the skeletal structure of *Siderastrea* *siderea*, a common massive Caribbean coral. Like *Porites*, *S. siderea* invests calcification resources in summer into linear extension^16^. This species of coral has been used in a wide range of paleoclimate studies^27–30^ owing to qualities such as their massive morphologies, wide distribution in the Caribbean, high longevity, resistance to bleaching^31^, and slow annual extension rates^32^. Two cylindrical cores, named Weston (W1) and Harrison’s Point (HP1), were extracted from two colonies growing on the north-west leeward coast of Barbados. W1 grew on a fringing reef at 5 m water depth near Weston and the other from a forereef setting at 15 m depth off the coast of Harrison’s Point. The skeletal growth banding was revealed through reconstructing intra-corallite porosity along the major growth axis by running an image segmentation algorithm on µCT images. This effective technique showed that the commonly observed growth pattern observed on radiographs originates from cyclic porosity variations in the corallite. Thus, throughout the manuscript we characterise the growth banding in terms of porosity rather than density (i.e., high porosity band is equivalent to low density band).

## Materials and methods

### Fieldwork and sample preparation

**Fig. 1:**
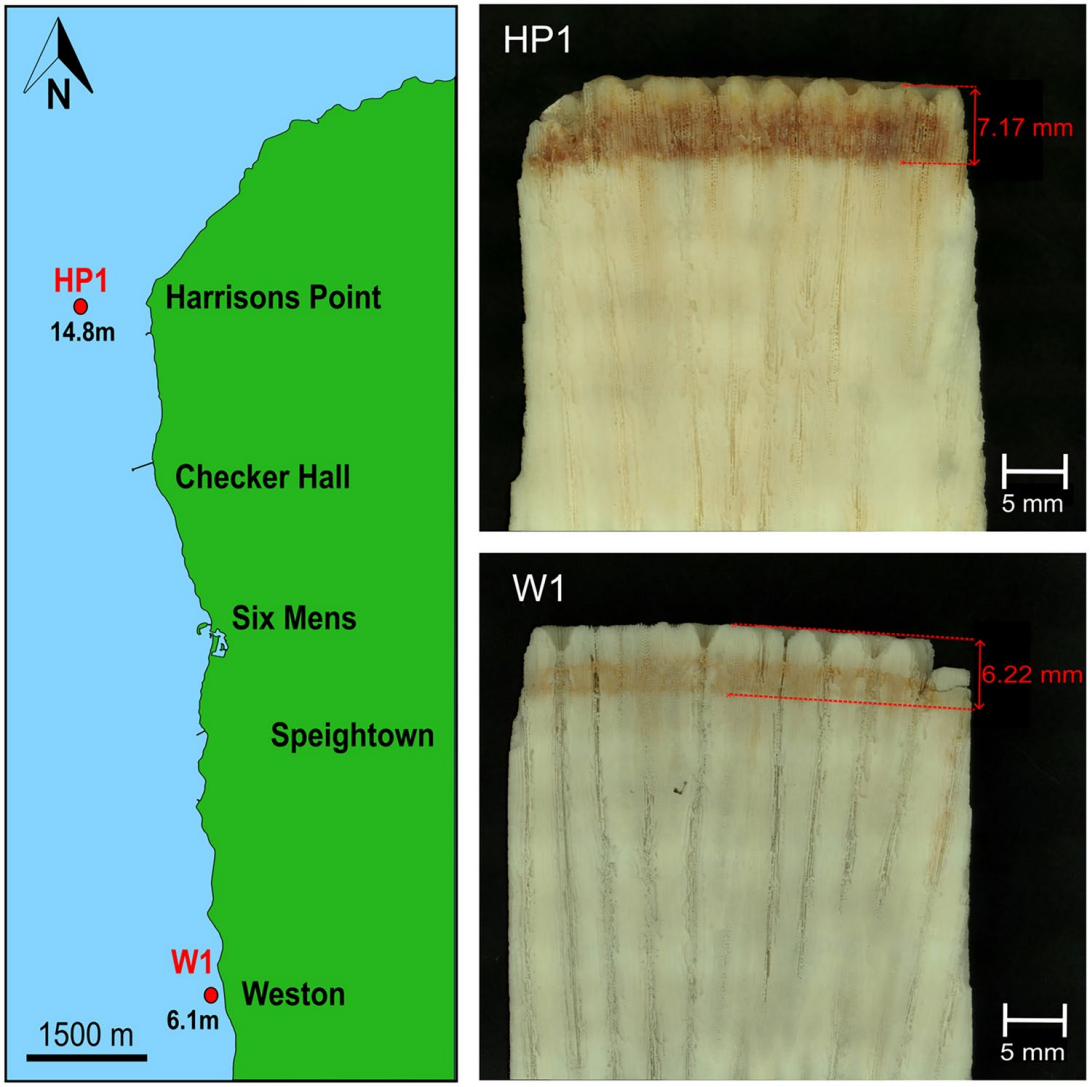
Left: Map of the north-west coast of Barbados showing the locations and depths of *S. siderea* samples HP1 and W1 (red circles). Right: Images of the major growth axis of HP1 (top) and W1 (bottom) illustrating the depth of soft tissue penetration (orange stain) at the growth surface. Note the thicker tissue thickness in HP1.

Two cylindrical coral cores, measuring between 6 and 6.5 cm in length and 4 cm in diameter, were extracted in July 2022 from two *Siderastrea siderea* colonies from the north-west coast of Barbados (Fig. 1). The first core was extracted from a colony growing on a forereef setting off the coast of Harrison’s Point (N 13°12.8844, W 059°38.5681) at a depth of 15 m and is hereon referred to as HP1. The second core was taken at a depth of 5 m from a colony growing on an (inshore) fringing reef, off the coast to Weston (N 13°12.8844, W 059°38.5681). This sample is hereon referred to as W1. Samples were collected via scuba diving using a pneumatic hand drill fitted with a 45 mm diameter, 50 mm long diamond-tipped core barrel. The drill was connected to an air-hose which attached to the quick coupling low-pressure inflator hose from a first stage scuba regulator. The scuba regulator in turn connected to a 12 L aluminium scuba tank via a DIN valve. A scuba buoyancy control device (BCD) was harnessed to the 12 L scuba tank and lead weights were fastened to the BCD set-up to keep the apparatus negatively buoyant during the extraction procedure. Once the cores were removed from the colonies, a brushed cement plug was inserted into the cavity to allow the corals to recolonise and recover. To remove organic tissue from the coral skeleton, the cores were directly inserted into a 1:3 solution of 7% active chlorine bleach and water for a minimum of 62 h, rinsed with fresh water, and left to dry at 25°C for 48 h. After bleaching, the depth of soft tissue penetration was evident through a band of dark orange staining at the outer surface (Fig. 1). The thickness of this orange band represents the depth at which soft tissues occupied the skeleton at the time of collection and was measured on the cores using a calliper. Each core was sectioned twice parallel to the major growth axis using a diamond-tipped saw at the Department of Earth Sciences, University of Geneva. The process aimed to 1) ensure that the growth direction was linear throughout the sample by medially partitioning each core into two semi-cylinders and 2) to remove the curvature of each semi-cylinder. The outcome of this procedure produced two skeletal slabs approximately 14 mm in thickness and 66 mm in length for each core. The slabs were inserted in deionised water and ultrasonically cleaned for a total of 20 minutes to remove cutting debris. The solution was changed every 5 minutes during sonication. The samples were left to dry at 45°C for 48 h and were stored in a screw-top container at room temperature.

### Micro-CT scanning

Micro-CT scanning (µCT) was performed on one slab from HP1 and W1 at the Haute École du Paysage, d’Ingénierie et d’Architecture (HÉPIA) in Geneva, Switzerland. The slabs were inserted separately into the scanner within a polystyrene holder. The holder was positioned on a rotating base, aligning the plane of x-rays parallel to the major growth axis. Both HP1 and W1 were scanned 4320 times helically over 3 complete turns, producing 16-bit TIFF images for each scan. X-ray emission voltage was 130 kV with a current of 61 mA. The size of the samples limited the voxel resolution to 29.2 and 25.4 µm for HP1 and W1 respectively. The volume graphic software “MyVGL” was used to visualise the three-dimensional volume of each sample µCT scans. To ensure we measured porosity variations in the plane of major growth, the three-dimensional reconstructions were adjusted using the MyVGL software to align the y-axis reconstruction plane parallel to the primary growth direction (i.e., corallite tracks). This ensured that each two-dimentional y-axis slice reconstructed was perpendicular to the major growth axis. The reconstruction generated 2254 and 2854 (16-bit TIFF) slices perpendicular to the growth direction for HP1 and W1. Due to the substantial volume of data generated by the reconstruction, an automated image processing approach was necessary to process the slices to assess skeletal growth.

### Image segmentation and classification

The methodology described below was developed to segment reconstructed x-ray slices from the µCT scans into pores and skeleton within classified skeletal microstructures (i.e., corallites and theca). Our approach is based on two fundamental steps:

(1) Primary pixel segmentation and classification of corallites and theca.

(2) Secondary pixel segmentation and classification of pores and skeleton.

**Fig. 2:**
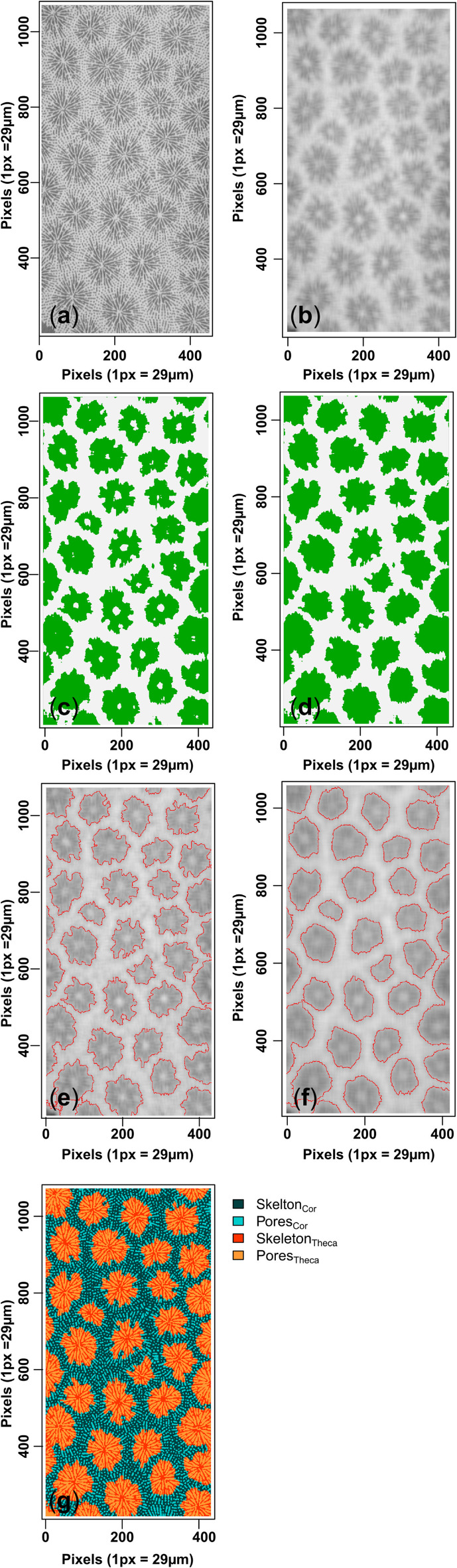
Visualisation of the image segmentation process on example slice #01127 from HP1. An area of the raw µCT slice is defined to avoid edge-related porosity effects (panel** a**). The area is smoothed using the pre-set S-value (i.e., S = 15 in panel **b**). The image segmentation algorithm is initiated on the smoothed slice (k = 2), clustering darker and lighter pixels into corallite and theca areas (green and white respectively in panel** c**). Internal clumps of lighter pixels (columella) are (mis)identified within the darker clusters (panel** c**) and are re-labelled as corallite (panel** d**). Panels **e** and **f** illustrate the effect of S (S = 15 and S = 25 respectively) on the corallite-theca boundaries. The area is then un-smoothed and the algorithm is reinitiated (k = 2) within the defined boundaries (i.e., in panel **e**) whereby red pixels correspond to corallite skeleton (Skeleton_Cor_), orange pixels to corallite pores (Pores_Cor_), dark blue pixels correspond to theca skeleton (Skeleton_Theca_) and light blue for theca pores (Pores_Theca_) in panel** g**.

**Fig.3:**
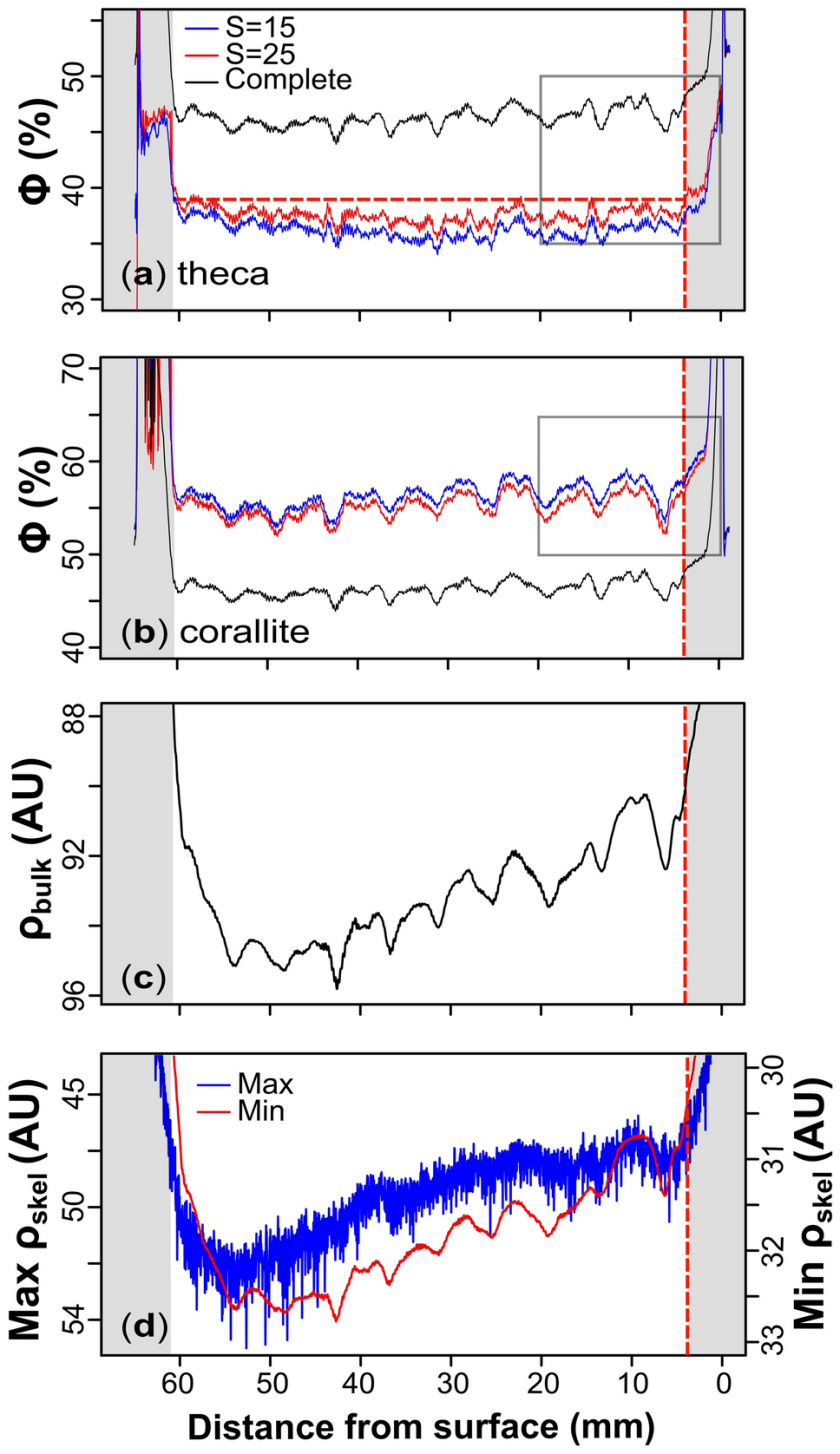
Graphs showing the reconstructed porosities of HP1 within the theca (panel** a**) and corallite (panel** b**) areas for both the larger (blue) and smaller (red) smoothing areas (S = 25 and S = 15 respectively) through the growth axis of the sample. Both graphs display the complete porosity reconstruction (i.e., combined theca and corallite porosities: black line) as a reference between the theca and corallite microstructures. The initial and terminal elevated porosities correspond to the edge-effected top and bottom surfaces of the sample (greyed-out). The 95^th^ percentile of values between the edge effect is illustrated by the horizontal, hashed red line (panel** a**). The grey box (panels **a** and **b**) corresponds to the growth surface porosity reconstructions in Figure 6a and 6c. The bulk skeletal density from the reconstructed radiograph of HP1 is shown in panel** c**. The bulk density reconstruction corresponds to the same area used in the porosity reconstructions in panels** a** and** b**. The density units are arbitrary as they represent the average of a one-dimension profile along the radiograph. The minimum (Min ρ) and maximum (Max ρ) skeletal density in panel** d** correspond to the greyscale values of pixels classified as skeleton (red and blue lines respectively). Note that the y-axis in panels** c** and** d** is inversed for better comparison to the porosity reconstructions.

**Fig. 4:**
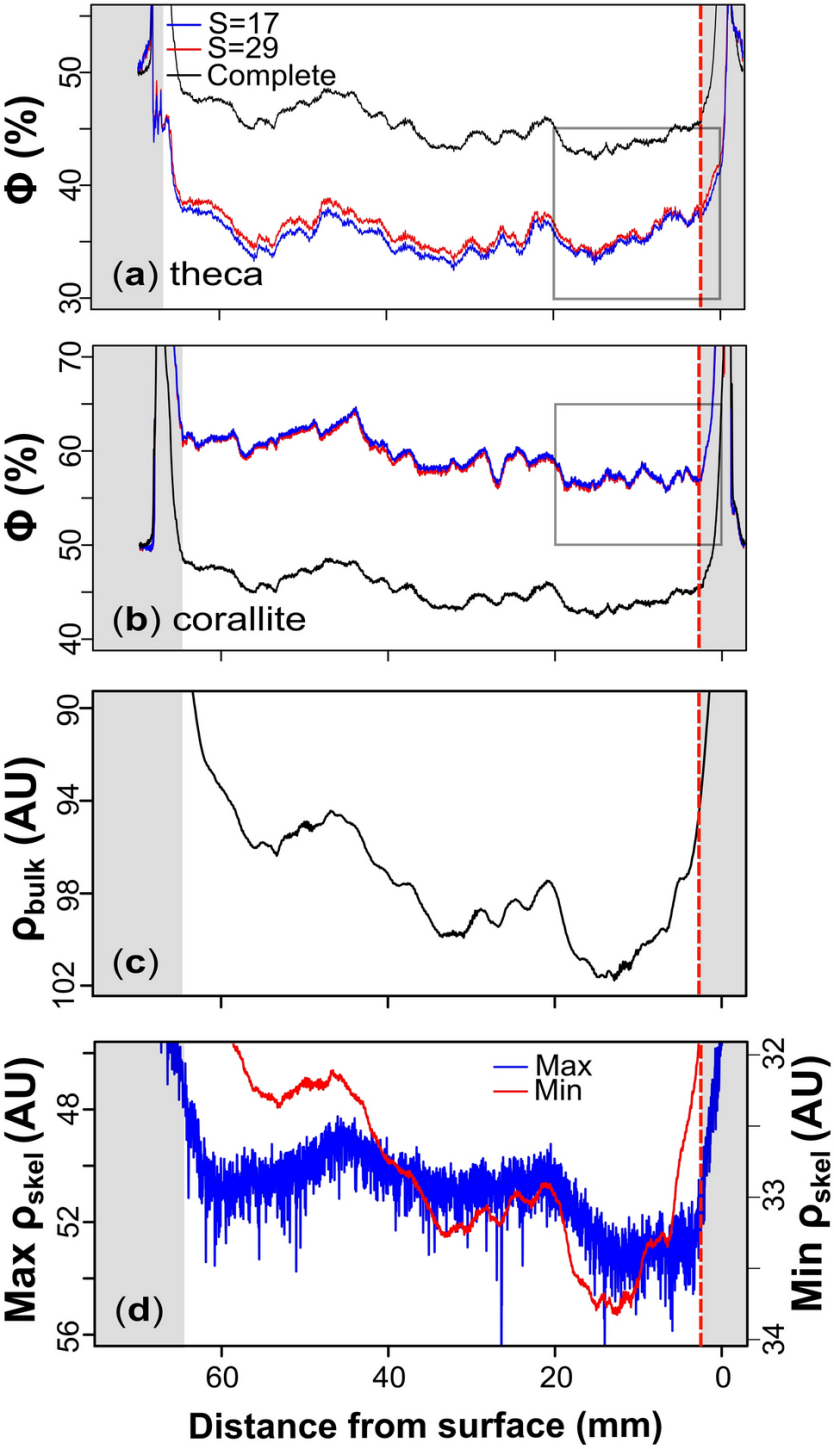
Graphs showing the reconstructed porosities of W1 within the theca (panel** a**) and corallite (panel** b**) areas for both the larger (blue) and smaller (red) smoothing areas (S = 29 and S = 17 respectively) through the depth of the sample. Both graphs display the complete porosity reconstruction (i.e., combined theca and corallite porosities: black line) as a reference between the microstructures. The initial and terminal elevated porosities correspond to the edge-effected top and bottom surfaces of the sample (greyed-out). The grey box (panels** a** and** b**) corresponds to the growth surface porosity reconstructions in Figure 6b and 6d. The bulk skeletal density from the reconstructed radiograph of W1 is shown in panel** c**. The bulk density reconstruction corresponds to the same area used in the porosity reconstructions in panels** a** and** b**. The density units are arbitrary as they represent the average of a one-dimension profile along the radiograph. The minimum (Min ρ) and maximum (Max ρ) skeletal density in panel **d** correspond to the greyscale values of pixels classified as skeleton (red and blue lines respectively). Note that the y-axis in panels **c** and **d** is inversed for better comparison to the porosity reconstructions.

The data required to assess porosity variability in coral skeletal structures are the reconstructed y-axis slices from the µCT scans. To avoid area-related bias associated with different pixel resolutions between HP1 and W1, the area (mm^2^) and its proportions defined on the slices for the image segmentation were kept the same at 323.6 mm^2^. The reconstructed slices display spherical to elliptical structures (corallites) that are predominantly composed of darker groups of pixels relative to the surrounding skeleton (theca) (Fig. 2a). The primary segmentation step exploits this difference in pixel greyscale between the light and darker groups of pixels to identify areas of the slice corresponding to corallite and theca. To exaggerate the difference in greyscale between the two groups, and to aid the segmentation, a 2D low-pass filter (i.e., mean average) was implemented on each slice using a uniform smoothing kernel with adjustable dimensions of S x S (Fig. 2b). This smoothing approach replaces each pixel with the average pixel value in a defined neighbourhood (S) of pixels. Smoothing was performed manually in R programming language^33^ as it allowed for better control of smoothing dimensions and allowed standardisation on how smoothing was achieved. Two smoothing conditions (S) were initiated on HP1: 15 and 25, corresponding to smoothing areas of 189.225 µm^2^ and 525.625 µm^2^, respectively. To account for the different voxel resolution in W1, S values of 17 and 29 (180.625 µm^2^ and 525.625 µm^2^, respectively) were chosen as the closest equivalents to the smoothing areas used in HP1. This comparison between the two smoothing parameters was important for determining how the degree of (two-dimensional) smoothing influenced the categorisation of theca and corallite (Fig. 2e-f) and subsequently the porosity reconstructions (Figs. 3a-b and 4a-b). On the smoothed image (Fig. 2b), a k-means clustering algorithm is performed to identify the two areas of pixels corresponding to corallites and theca depicted in Figure 2c. Two post-processing steps are performed to ensure that corallites are correctly segmented from the theca. Firstly, to overcome issues related to new corallite formation, a minimum clustering area was pre-defined (0.31 mm^2^) in the algorithm conditions to exclude newly formed corallites until they exceeded this critical size. Secondly, for larger corallites, the columella region was sometimes identified as theca (white spaces in the corallite area in Fig. 2c) and was therefore re-labelled as corallite (Fig. 2d). This was performed automatically by identifying regions of [incorrectly] labelled theca completely enclosed by corallite pixels. The outcome of the primary segmentation step on the smoothed slice classified the slice into (pixel) areas corresponding to either the corallites or theca (Fig. 3e-f). The second segmentation step reinitiates the k-means algorithm (k = 2) within the theca and corallite areas on the original (unsmoothed) slice to classify pixels as either pore or skeleton in the corallite and theca areas (Fig. 2g). Given that this method clusters darker and lighter pixels based on pixel greyscale, the resulting reconstructions represent variations in the ratio of pixels clustered as pores and skeleton. It is thus more appropriate to use “porosity” rather than density when describing the results. We present the porosity (in %) for: (i) the complete skeleton (Eq. 1); (ii) the theca (Eq. 2); and (iii) the corallite (Eq. 3).

1 $$\Phi_{Complete} = \, \left( {Pores_{Cor} + \, Pores_{Theca} } \right) \, / \, \left( {Area_{Cor} + \, Area_{Theca} } \right) \, * \, 100$$

2 $$\Phi_{Theca} = \, \left( {Pores_{Theca} / \, Area_{Theca} } \right) \, * \, 100$$

3 $$\Phi_{Corallite} = \, \left( {Pores_{Cor} / \, Area_{Cor} } \right) \, * \, 100$$

### Tracking density variations

To assess the correspondence between the growth banding pattern observed in traditional radiographs and variations in skeletal porosity, we reconstructed a traditional radiograph using the two-dimensional y-axis slices from the µCT scans of HP1 and W1. For each slice in the vertical orientation of the core, we integrated the greyscale values over: (i) the segmented area in section "Image segmentation and classification", and (ii) the total slice. This produced a radiograph image parallel to the axis of major growth for HP1 and W1 (see Supplementary Figure S1). To explore density variations in more detail, we calculated the minimum and maximum greyscale values for pixels classified as skeleton in the previous part of the method. In this study, each voxel is characterised by a greyscale value that represents x-ray attenuation. The greyscale values are not standardised and thus cannot be compared between different scans. Hence, we use arbitrary units to show relative changes in skeletal density.

## Results

### Large vs small smoothing area effects on corallite-theca boundary

The two smoothing kernels that were used to compare how smoothing influences the classification between corallite and theca are shown in Figure 2e-f. In the raw, un-smoothed µCT slices (Fig. 2a), darker pixels (i.e., pores) are shown to protrude from the corallite into the theca. Smoothing these pixels with the smaller area for HP1 preserves these protruding darker pixels at the edge of the corallites. Consequently, the segmentation algorithm distinguishes these darker pixels from the theca pixels and categorises them into the corallite area which resulted in more irregular corallite boundaries (Fig. 2e) than in the larger smoothing area (Fig. 2f). In contrast, implementing the larger smoothing area effectively blurs the apexes of the protruding darker pixels into the surrounding theca. Consequently, the segmentation algorithm classifies these pixels and the surrounding whiter pixels (i.e., theca) into the corallite area and also into the theca area (Fig. 2f). This produces more rounded, elliptical corallites in Figure 2f.

The porosity reconstructions obtained from the two smoothing areas for HP1 (S = 15 vs S = 25; Fig. 3a-b) and W1 (S = 17 vs S = 29; Fig. 4a-b) reveal similar smoothing-related differences in the reconstructed porosity. Both samples display a relatively higher mean corallite porosity when smoothing over the smaller area than smoothing over the larger area (see Table 1). Smoothing has an inverse effect on theca porosity in both samples, with the larger smoothing areas yielding higher theca porosities compared to smaller areas (see Table 1). A smaller S-value better captures local regions of porosity extending from the corallites into the cluster, leading to increased corallite porosity (Figs. 3b, 4b) and decreased theca porosity (Figs. 3a, 4a). In this context, the larger smoothing area is also shown to merge/join multiple corallites (Fig. 2f) which consequently contributes to the decrease in corallite porosity as relatively more whiter pixels (i.e., skeleton) are incorporated into the corallite area. Continuously increasing the S-value decreases the corallite area until the algorithm can no longer distinguish between the two (theca and corallite) clusters. At such point, the porosity signal between the theca and corallite converges (see Supplementary Figure S2). In contrast, decreasing the smoothing area causes the corallite and theca porosity signals to diverge.

**Table 1:** Summary of image segmentation results for HP1 and W1 under two values of S.

Sample	Microstructure	Smoothing (S)	Porosity (%)	Mean Area (mm^2^)
W1	Corallite	17	59.8	127.7
W1	Theca	17	35.5	182.7
W1	Corallite	29	59.4	119.7
W1	Theca	29	36.2	179.5
HP1	Corallite	15	56.3	155.3
HP1	Theca	15	36.2	155.0
HP1	Corallite	25	55.2	148.9
HP1	Theca	25	37.5	150.6

This table shows the mean porosities of the theca and corallites microstructures, as well as the mean corallite area for HP1 and W1 under the small and large smoothing areas (S). All calculations exclude the edge-effected slices defined in the text in section "Porosity signal".

Regardless of the S-value, the algorithm consistently captures the same relative patterns in both the corallite and theca porosity. The correlation coefficient between the corallite porosity reconstructions when smoothed over the smaller and larger area was r = 0.98 for HP1 (Fig. 3b) and r = 0.98 for W1 (Fig. 4b), implying that differences in smoothing does not modify porosity patterns.

### Porosity signal

The results of the segmented µCT porosity reconstructions for HP1 and W1 are displayed in Figures 3a-b and 4a-b. Each reconstruction consists of three porosity signals representative of: (i) the complete area (black); (ii) the corallites (red); and (iii) the theca (blue). The initial and terminal stages of the porosity reconstructions are characterised by elevated and highly variable porosities that correspond to the top and bottom surfaces of the sample. Here we find irregular, uneven surfaces resulting in partially filled µCT slices containing empty spaces (darker pixels) which are responsible for yielding higher porosities. We term this phenomenon ‘edge-effect’ as it effects the top and bottom edges of the sample. The top growth surface of the sample also contains the hollow corallite structures, due to the depression in which the coral polyp sits. These cup-like morphologies form multiple depressions on the sample surface, creating a decreasing gradient in porosity until the plane of reconstruction is parallel to the depth of the columella/base of the cup. This decreasing gradient in porosity is most clearly displayed in the corallite porosity of HP1 (Fig. 3b). The theca also displays a growth surface decrease in porosity (Fig. 3a). These observations are also observed in W1 (Fig. 4a-b). At the base of the skeletal sample, an irregular, uneven surface exists where the core was not broken perfectly perpendicular to the growth direction. This also contributes to the edge-effect and consequently to the relatively higher porosities.

Between the edge-effected intervals of heightened and variable porosity exists a more consistent porosity signal. In the case of HP1, nine porosity cycles are clearly observed between 4 and 60 mm from the skeletal surface which are prominent in both the complete and corallite areas. This cyclicity is noticeably absent in the theca area, which displays a relatively more constant porosity signal. In HP1, the mean porosity for the complete, corallite and theca areas between the edge-effected slices (with the larger S = 25 smoothing area) is 46.3, 55.2, and 37.5 % respectively. The clearest cyclical porosity signal is recorded in the corallite area (i.e., porosity changes within all corallite areas per slice), which evidences irregular distances between cycles. The troughs were chosen to measure the linear distance between each cycle owing to their clarity and were located using the minimum value between the approximate location of the peaks of each cycle. The resulting inter-trough distance ranged from 5.08 to 7.36 mm, with a mean of 6.04 mm. The correlation coefficient between the porosity of the corallite (red line Fig. 3b) and the complete porosity of HP1 (black line in Fig. 3b) reveals a strong positive correlation (r = 0.87) compared to the theca (r = 0.54). No relationship is observed between the theca and corallite porosity in HP1 (r = 0.21).

The porosity signal between the edge-effected slices in W1 is recorded between 2.65 and 63.94 mm along the major growth axis of the sample. The reconstruction from this shallower sample shows higher porosity variability and subsequently less clear cyclicity than in HP1, making it difficult to determine the number of porosity cycles. The mean porosities for the complete, corallite and theca areas with the larger smoothing area are 45.4, 59.4 and 36.1 %, respectively. A general decrease in porosity is observed throughout the reconstruction, decreasing towards the growth surface. Although the exact number of cycles is uncertain, there seems to be more cycles than in HP1. Unlike HP1 however, the theca porosity reconstruction displays two orders of cyclicity: 1) relatively small cyclical patterns (approximately 4 mm wavelength) superimposed on 2) three longer-wavelength cycles (approximately 22.9 mm in wavelength). The correlation coefficient between corallite porosity (red line in Fig. 4b) and the complete porosity (black line in Fig. 4b) (r = 0.90), as well as the theca porosity (red line in Fig. 4a) and the complete porosity (r = 0.83) exhibit strong positive correlations. A moderate positive correlation also exists between the theca and corallite porosity (r = 0.55).

### Radiographs

The skeletal density from the reconstructed radiographs (Supplementary Figure S1) of the major growth axis of HP1 and W1 are shown in Figures 2c and 3c respectively. To ensure that the areas defined in the porosity reconstructions capture the density variations of the entire samples, the bulk density of the total area of the radiograph was compared with the bulk density of the area used in the segmentation algorithm. This comparison reveals a strong positive correlation for both HP1 and W1 (r = 0.99 – Supplementary Figures S3 and S4), and so hereafter we focus on the bulk density change only in the segmented area. The bulk density reconstructions of HP1 and W1 both show low and variable densities related to the edge-effected slices at the initial and terminal stages of reconstructions (grey boxes in Figs. 3c, 4c).

In comparison to the porosity reconstructions of HP1, skeletal density is non-stationary and is shown to decrease towards the growth surface (Fig. 3c). Comparing the bulk density (Fig. 3c) of HP1 to the minimum and maximum greyscale values in skeletal pixels (red and blue lines in Fig. 3d respectively) reveals strong positive correlations (r = 0.97 and r = 0.86 respectively). Despite the non-stationary bulk density signal in HP1, eight cycles are observed. These bulk density cycles are inversely correlated with the complete porosity from the segmentation algorithm (r = -0.79).

Similar to the porosity reconstructions of W1, cyclicity in the bulk skeletal density is less clear than in HP1. The bulk density of W1 (Fig. 4c) is moderately inversely correlated with the complete porosity (r =- 0.62 – black line in Fig. 4a-b). The bulk density of W1 is observed to increase towards the growth surface. Comparing the bulk density (Fig. 4c) to the minimum and maximum greyscale values of skeletal pixels (red and blue lines respectively in Fig. 4d) reveals a strong positive correlation (r = 0.98 and r = 0.89, respectively).

### Corallite area and microstructure components

**Fig. 5:**
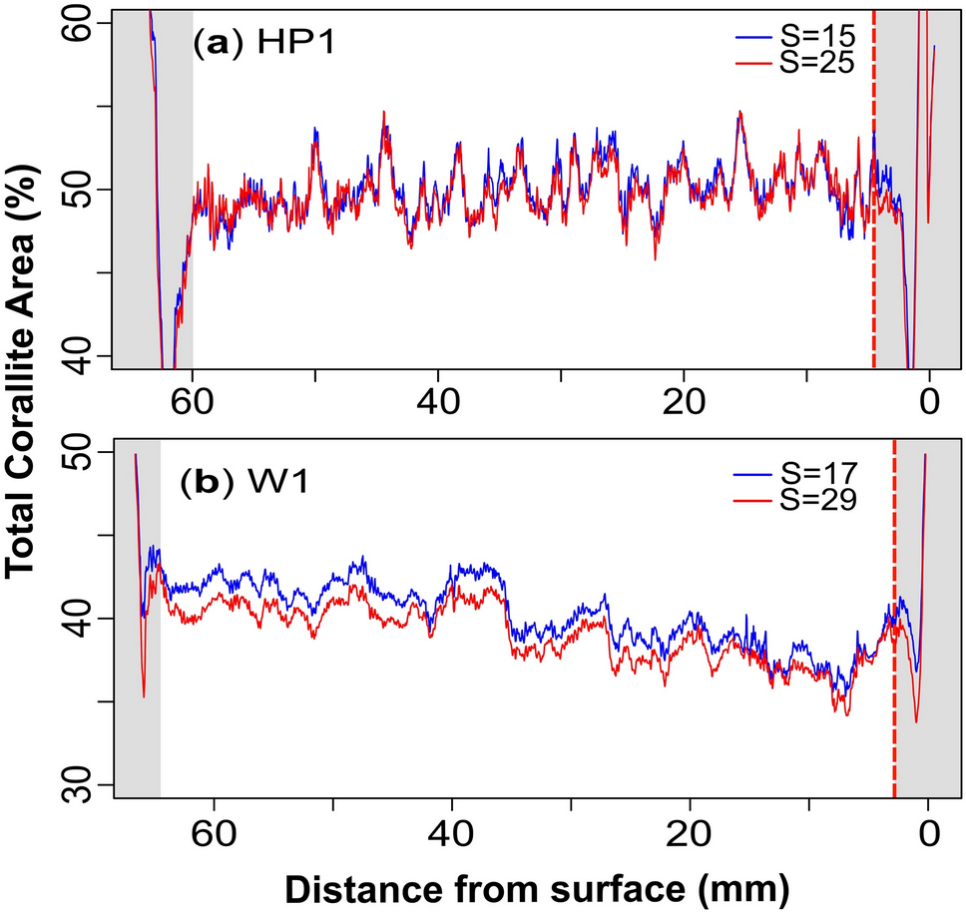
Graphs showing the total mean corallite area through the major growth axis of HP1 (**a**) and W1 (**b**) when smoothed over small and large areas (blue and red lines, respectively). The variability of the total corallite area in HP1 shows a stationary signal. In contrast, the total corallite area in W1 reveals relatively longer-term variability and a general decreasing signal towards the growth surface. Note that the different smoothing areas in HP1 causes little offset between corallite area (red and blue lines in panel **a**) whereas in W1, smoothing diverges the two area reconstructions (panel** b**). The greyed-out box represents the growth surface edge effect in each panel.

The percentage of corallite area in the total area (i.e., theca and corallite areas combined) of HP1 ranges from 45.8 and 54.7 % (Fig. 5a). Although the corallite area shows variability, the mean corallite area (49.8 %) remains stationary throughout the reconstruction. There is a strong positive correlation between the two classes of pixels in the corallite (skeletal and pore pixels) and the corallite area fluctuation, with pores showing a correlation of r = 0.80 and skeleton with r = 0.71. From these classes, the variation in corallite pores is positively correlated to the variation in complete porosity (r = 0.77) and the variation in corallite skeleton is weakly negatively correlated to the variation in complete porosity (r = -0.39). In contrast, the classes (i.e., pores and skeleton pixels) in the theca presents an inverse relationship with corallite area, where pores have a correlation of r = -0.85 and skeleton r = -0.93. The variation of both classes in the theca, however, shows no correlation between the complete porosity and pores: (r = 0.05), but a moderate negative correlation with the skeleton (r = -0.51). Additionally, no significant relationship (r = 0.31) exists between the variation in corallite area (Fig. 5a) and the complete porosity variation (black line in Fig. 3a-b) in HP1.

The percentage of corallite area in the total area of W1 varies between 35.1 and 44.2 % with a mean corallite area of 40.0 % (Fig. 5b). There is a 9.9 percentage-point decrease in the mean total corallite area in W1 towards the growth surface of the sample. A strong correlation is observed between the variation of pores (dark pixels) in the corallite and the fluctuation in mean corallite area (r = 0.93). In contrast to HP1, there is no relationship between skeleton pixels in the corallite (light pixels) and the total corallite area (r = 0.23). The pores in the corallite are strongly correlated to the complete porosity fluctuation in W1 (r = 0.86), whereas the skeleton in the corallite area is much less correlated (r = -0.43). Similarly to HP1, an inverse correlation exists between the skeleton in the theca and the total corallite area (r = -0.85). A weak inverse relationship is noted between the pores in the theca and the total corallite area (r = -0.42). There is no relationship between the pores in the theca and the complete porosity in W1 (r = 0.3), but a strong relationship exists between the skeleton variation in the theca and complete porosity (r = -0.93). There is also a moderate positive correlation (r = 0.67) between the variation in the corallite area (Fig. 5b) and the complete porosity variation (black line in Fig. 4a-b) in W1.

## Discussion

### Porosity driven density variations

The strong positive correlations between the complete porosity and bulk density reconstructed from the µCT scans implies that density banding in *S. siderea* is driven by porosity variations. Based on the Nyquist-Shannon theorem^34^, the sample-size restricted voxel resolution means that skeletal structures or pores under 58 and 50 µm for HP1 and W1 are not fully resolvable. Therefore, the segmentation algorithm used to reconstruct porosity tracks relatively larger open spaces such as inter-septal porosity in the corallite. At the resolution of the scans, each slice can be split into two main pixel components: skeleton and pores. The distinction of these two components is based on the greyscale values reconstructed from the micro-CT scans. Nevertheless, a pixel may fall onto a boundary between these two components or may include a mixture between large pores and unresolvable microstructures, or skeleton and unresolvable pores (or other components such as organics). Nevertheless, when we reduce the resolution and induce more mixing between components (Supplementary Figure S5), we still record the porosity variations observed in Figures 3–4.

Longer-term variations in bulk density, however, do not follow the reconstructed porosity, especially for sample HP1. To understand this, we compare the bulk density (Figs. 3c, 4c, respectively) to the minimum and maximum greyscale values for pixels classified as skeleton (red and blue lines in Figs. 3d, 4d, respectively). Firstly, we observe that the maximum values follow the same long-term trends observed in the bulk porosity for W1 and HP1. As the maximum value represents pixels containing the most skeleton, any changes in this value can only be due to mixing with an unknown lower density component smaller than the resolution of the scan, which we interpret to be microporosity. Other components such as organics would take the space of microporosity (thus increasing the greyscale value) and so would require additional microporosity to reduce this maximum greyscale value.

The observed cyclicity in the bulk density and porosity is present in the minimum greyscale value of pixels classified as skeleton in HP1, with high density correlated with low porosity. Therefore, we interpret the variation in the minimum greyscale value as representing variations in unresolvable nano/micro porosity that follows the reconstructed porosity changes observed in intra-corallite spaces. Hence, although this microporosity in unresolvable visually in the µCT reconstruction, we are still able to track relative changes throughout the samples.

### Skeletal thickening interval

**Fig. 6:**
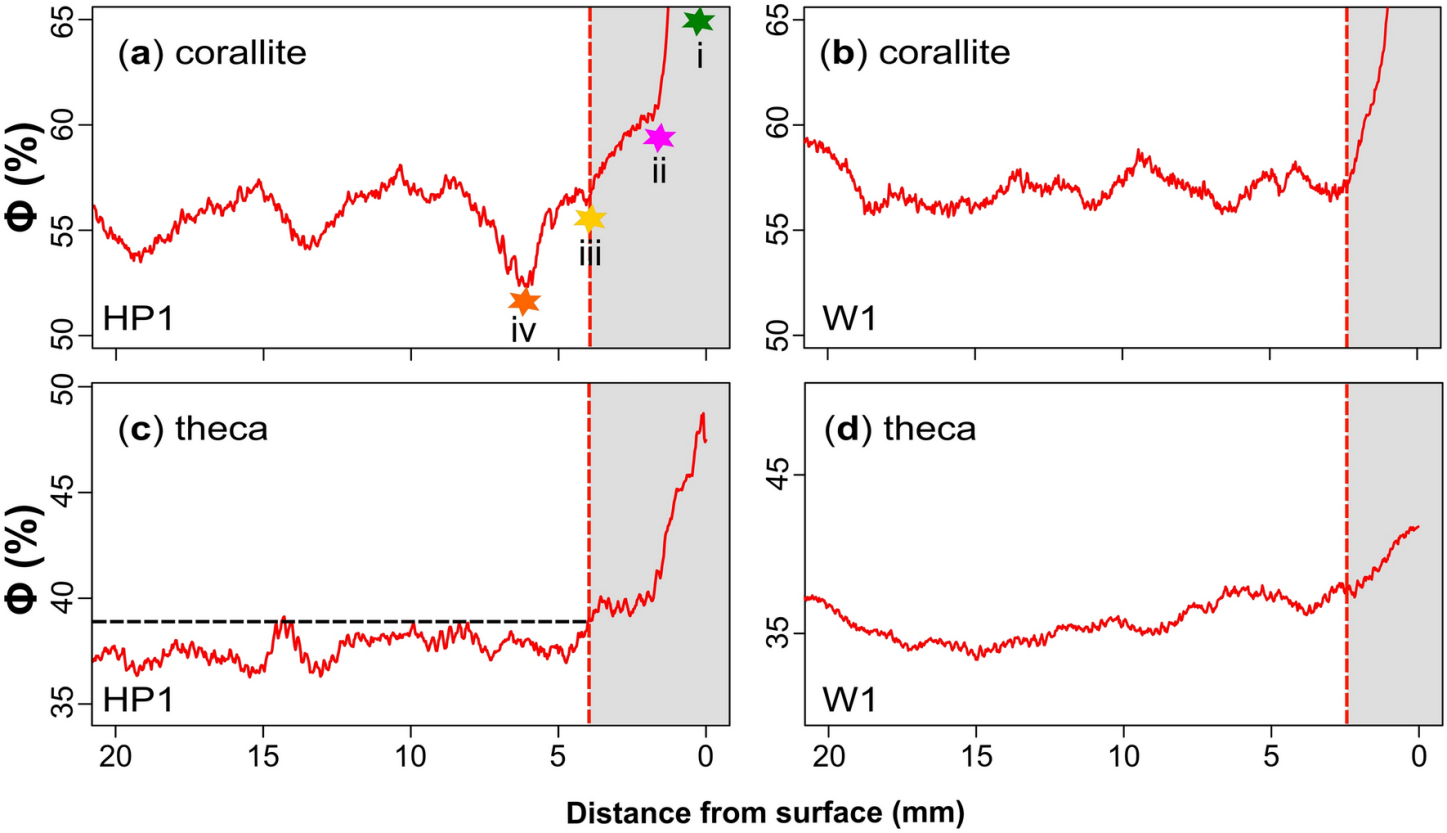
Corallite and theca surface porosity profiles for HP1 (panels** a** and** c** correspond to the grey boxes in Fig. 3a-b, respectively) and W1 (panels** b** and** d** correspond to the grey boxes in Fig. 4a-b, respectively). The coloured stars (i–iv in panel **a**) are used to illustrate the key depths in which porosity is reconstructed within the corallites of HP1. The green star (i) marks the growth surface. The pink star (ii) corresponds to the depth in which the plane of reconstruction reaches the bottom of corallite-cups. The transition between the pink and yellow star (ii–iii) correspond to the depth in which skeleton is thickened beneath the corallite cup. Skeletal thickening is also observed in the theca porosity profile (panel** c**). The vertical, red hashed line (panels** a** and** c**) correspond to the same thickening depth. The horizontal dashed black line in the theca reconstruction (panel** c**) represents the 95^th^ percentile of theca porosity between the edge-effected slices (greyed-out box). The yellow star (iii in panel **a**) marks the most recent growth band formation at the time the sample was collected. The orange star (iv) corresponds to the winter HDB. Although less clear, these porosity transitions in W1 at the growth surface is evident in the corallite and theca porosity profiles (panels** b** and** d** respectively).

The porosity variability in both the corallite and theca areas at the growth surface of HP1 and W1 indicates that skeletal thickening processes occur across both microstructures. The growth surface of HP1 and W1 reveals porosity transitions from high porosities attributed to edge-effected slices to relatively lower porosities which fall in line with pre-existing porosity trends (Fig. 6). The observation that thickening is present in both microstructures is generally supported by previous literature on other massive corals such as *Porites *^1,21^, which state that thickening occurs throughout the total depth of the CTT, which encapsulates both microstructures. Thickening across both microstructures throughout the soft tissues in *S. siderea* is also reported in literature^10^, whereby the thickness of the theca, columella and septa is reported to vary along with the size and arrangement of synapticulae, and the spacing of dissepiments^35,36^. Dissepiments have been reported to vary one-to-one with lunar cycles for massive *Porites*^3^ which has a similar growth strategy to *S. siderea*^16^. Such structures and associated intra-skeletal porosity may contribute to the density variations shown in typical radiographs (see Supplementary Figure S1) and the reconstructed density in Figures 3c and 4c. This is discussed in section "Growth banding". The depth and infilling (i.e., thickness) of the corallites in *S. siderea* were also reported to vary in response to pH and thermal stress^37^, highlighting the sensitivity of skeletal thickening processes to environmental parameters.

Due to the relatively slower extension rate and stronger edge-effects in W1 (see Supplementary Figure S6), distinct transitions in porosity are more apparent in the upper skeleton of HP1 (Fig. 6a, 6c). Considering that the sample was extracted in July 2022, which is approximately two months prior to the annual thermal maximum typically in September, we interpret that the porosity trough closest from the coral’s growth surface (orange star (iv) in Fig. 6a) as the previous thermal minimum in March. This logic dictates that the low porosity bands (i.e., HDB) in HP1 correspond to relative cooler SSTs in the dry season (Dec – May) whilst the high porosity bands (i.e., LDB) are formed during relatively warmer SSTs in the wet season (Jun – Nov). This observation is supported by literature which report that *S. siderea* has a similar growth strategy to massive *Porites* which increases linear extension rates and decreases skeletal density with increasing SST (i.e., calcification rates)^16^.

**Fig. 7:**
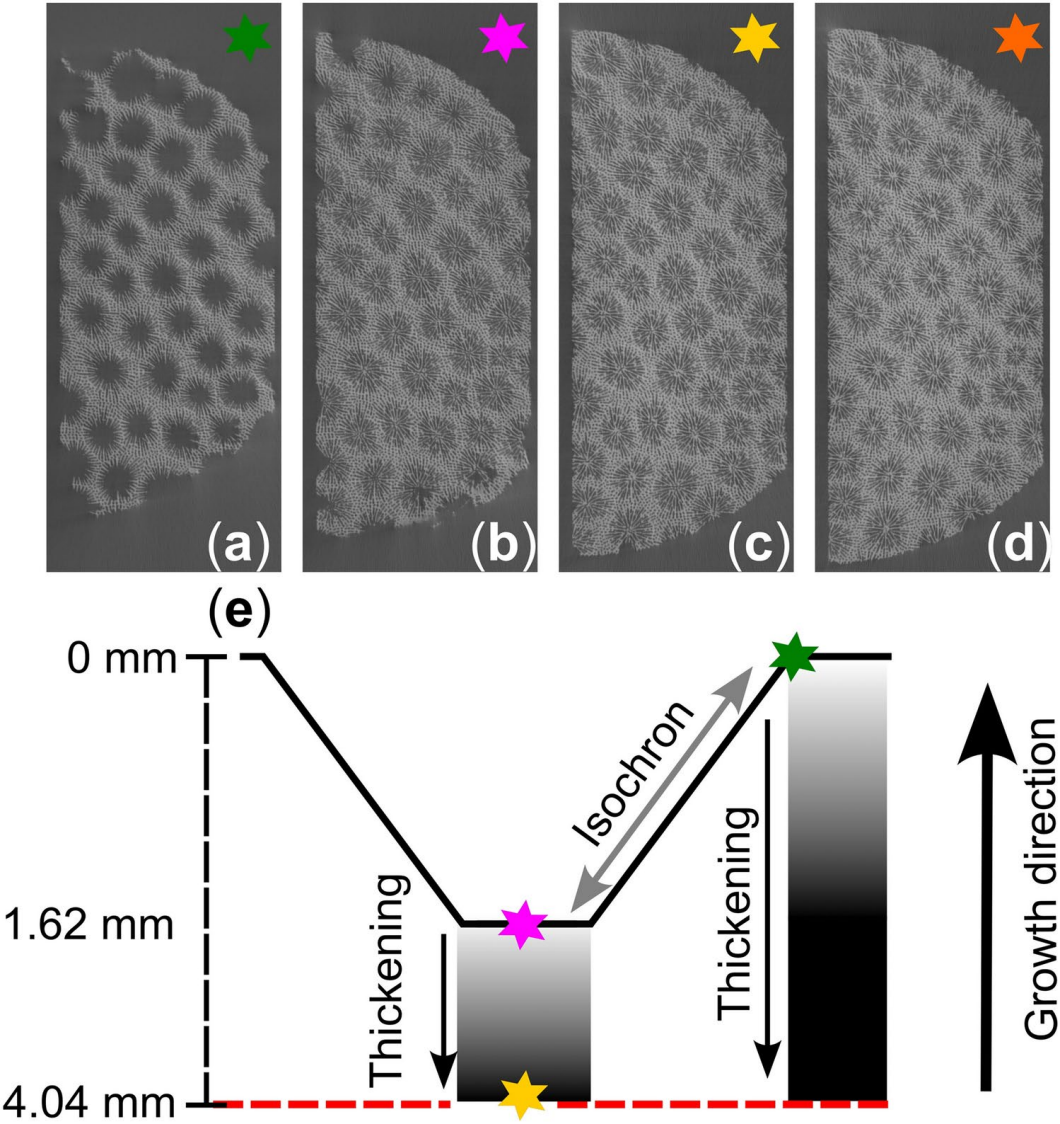
Four µCT slices (**a**-**d**) correspond to the corallite thickening interval illustrated by coloured stars in Figure 6a. A schematic representation (**e**) reconstructs the thickening stages which are illustrated by the same coloured stars with depth. The horizontal red hashed line (panel **e**) corresponds to the vertical red hashed line in Figure 6a, 6c. The higher porosities exhibited at the start of the reconstruction correspond to partially filled/hollow corallites and uneven surfaces (green star in panels** a** and** e**). The corallite microstructures are reconstructed at a 1.56 mm depth whereby the porosity cyclicity begins (violet star in panels** b** and** e**). The corallite microstructures are thickened between 1.56 to 4.35 mm depth which correspond to the environmental conditions at the time of sample extraction (yellow star in panels** c** and** e**). The porosity minima (orange star in panel** d**) corresponds to the previous winter.

The theca and columella microstructures are biomineralised at the same time and under the same environmental conditions yet are spatially separated vertically in the coral skeleton. This spatial difference arises from the morphology of the corallite cup structure and consequently forms an isochron between the top of the theca (i.e., rim of the cup) and the top of the columella (i.e., base of the cup) (Fig. 7e). Consequently, each two-dimensional slice is affected by this isochron and has implications when comparing different microstructural porosities on the same depth scale. This cup morphology is evidenced by the elevated porosities close to the growth surface within the corallite area due to partially filled, hollow corallites in the µCT slices (green star (i) in Figs. 6a, 7a,7e). Close to the growth surface, the plane of reconstruction (slice) initially skims the growth surface (i.e., theca) of the sample and contains hollow depressions and no corallite microstructures (green star (i) in Figs. 6a, 7a, 7e). As the plane of reconstruction moves further into the sample, the internal structures of the corallite (i.e., septa) are gradually reconstructed and consequently the porosity decreases until the horizontal plane reaches the bottom of the cup (columella – pink star (ii) in Figs. 6a, 7b, 7e). At approximately 1.62 mm below the growth surface, the porosity signal corresponds to the filled corallites on the slice (pink star (ii) in Figs. 6a, 7b, 7e) and represents the average depth of the corallite cups close to the growth surface. From the base of the corallite cup (i.e., columellae), the corallite porosity signal decreases from 60.9 % to 56.4 % until 4.04 mm below the growth surface (pink to yellow star (ii-iii) in Figs. 6a, 7b, 7c, 7e). At 4.04 mm beneath the growth surface, the porosity is in alignment with the upward slope of the preceding summer growth cycles. This is consistent with the temporal context of the sample which was collected in early summer (July) during the formation of the high porosity band. We suggest this is where newly extended skeleton in the corallite region was thickened within the soft tissues at the time of core extraction, with this process visible on the raw µCT slices (pink to yellow star (ii-iii) in Fig. 7b-c). This is shallower than the total depth of the tissue layer at extraction (7.17mm; Fig. 1) suggesting that skeletal thickening is not happening throughout the total tissue layer but rather focussed in the upper two-thirds of the soft tissues. The low porosity (i.e., HDB) corresponding to winter 2022 is shown at 6.02 mm below the growth surface (orange star (iv) in Fig. 6a) and is visible on the raw µCT slice in Figure 7d. Although the corallite growth surface porosity profile of W1 is less clear, both microstructures show a similar thickening interval within the upper part of the tissue layer, from the growth surface to approximately 2.65 mm (red dashed line in Fig. 6b, 6d).

The theca porosity profile of HP1 at the growth surface (Fig. 6c) however shows that thickening occurs directly from the growth surface to 4.04 mm depth, at which point the porosity reaches the 95th percentile of its distribution (black dashed line in Fig. 6c). This profile also shows a decrease in porosity from 48.8 to 41.1 % (7.7 point-percent), from the growth surface to 1.62 mm (the depth of corallite base). From 1.62 to 4.04 mm, the same depth interval in which the corallite is thickened, the decrease in porosity in the theca is significantly less (1.3 point-percent) which suggests a reduction in the rate of thickening in the theca. For the same depth interval in the corallite area however, a 4.4 point-percent decrease in porosity is observed, suggesting that the focus of calcification has shifted from the theca to the corallite region within the same interval of soft tissues. These findings suggest that whilst both structures are thickened to similar depths in the soft tissues, the rate of thickening between microstructures and within the soft tissues is not the same. The difference in rate of thickening between different microstructures may be explained by the work of Chalk et al., ^38^ who propose that, for *S. siderea,* the skeleton does not precipitate from a spatially homogeneous fluid. The calcifying fluid in the corallite region is upregulated in [HCO_3_^-^] ^39^ and dissolved inorganic carbon (DIC) ^40^, which are both crucial for controlling calcification ^41–44^. In contrast, the calcifying fluid in the theca region is upregulated in pH, [CO_3_^2-^], aragonite saturation state (Ω_ar_) ^38,45–48^, as well as [HCO_3_^-^]. Importantly, the elevated Ω_ar_ in the theca region favours the precipitation of new crystals and increases theca density ^45,49^. Although further analyses and additional samples are required, the research discussed above in relation to our findings suggests: 1) that upregulation of pH, [CO_3_^2-^], Ω_ar_, and [HCO_3_^-^] in the theca is concentrated between 0 and 1.62 mm and 2) the upregulation of DIC and [HCO_3_^-^] in the corallite region is focused between 1.62 and 4.04 mm below the uppermost growth surface of the coral.

Unlike previous studies which state that skeletal thickening occurs throughout the total depth of the tissue layer^1,13,17,20,21^, our results show that skeletal thickening is focused within the top two-thirds of CTT, and that the rate of thickening varies between microstructures within soft tissues. These findings question the role of CTT in the timing of porosity band formation, which is reported to vary in response to environmental parameters (i.e., water quality and nutrients) and throughout seasonal growth^1,50–53^. Figures 3b and 6a show that growth banding in HP1 is recorded in the corallite region. Assuming that the corallite thickening interval (1.62 to 4.04 mm) is proportionate to the CTT, and that the formation of growth bands is driven by CTT and extension rates^21^, variations in the CTT throughout coral growth will influence the vertical distance between the growth surface (theca) and the growth band, complicating interpretations.

An important limitation to consider when interpreting the corallite porosity at the growth surface is the effect of variable skeletal extension rates. The growth surface of massive coral colonies is often irregular/uneven as a result of non-linear skeletal extension rates across different parts of the colony. In such cases, the plane of reconstruction at the growth surface crosscuts corallites at different depths and consequently different stages of skeletal thickening. The yielded porosity signal therefore represents an average of multiple corallites at different stages of thickening, smearing the pristine thickening signal. Non-linear extension can have a similar effect deeper into the skeletal archive, whereby past irregular/uneven growth surfaces may make it challenging to reconstruct an accurate porosity signal. For HP1, we assume that the corallites extended linearly due to the homogenised porosity cyclicity between corallites, and the clear distinctions between the porosity transitions at the growth surface. In W1 however, there is higher uncertainty in the exact position of coral thickening due to the uneven growth surface of W1. The unevenness of the growth surface is observed on the reconstructed slices and reconstructed radiography (Supplementary Figures S6 and S1) which show areas of corallites that are elevated relative to other parts. Therefore, the two-dimentional plane of the slice at the surface will display corallites in elevated regions before corallites in depressed regions. The unevenness of the growth surface consequently means that slices cut corallites at different depths and consequently at different stages of cup formation and thickening. The result of this effectively blurs the corallite morphology and thickening signals in porosity which are preserved in HP1. Despite the higher uncertainty in the depth of coral thickening and the relatively poorer clarity porosity signal at the growth surface of W1, the relative porosity variations at the growth surface across both microstructures remain accurate. To address non-linear growth patterns in future studies, it is advisable to target colonies without visible surface defects and larger colonies due to their reduced surface curvature, which offers a more conducive geometry for analysis. Alternatively, reconstructing and tracking singular corallites through the depth of the skeleton can provide a more precise method of averaging the thickening signal of multiple corallites in a colony, helping to remove non-linear growth effects.

### Growth banding

Comparing corallite porosity to the bulk porosity of HP1 reveals a stronger positive correlation (r = 0.87) compared to correlating theca porosity to the bulk porosity (r = 0.54). This suggests that while porosity variations are present in both the theca and corallite in HP1, fluctuations in corallite porosity dominate the bulk porosity signal (black line in Fig. 3a-b). The negative correlation between the corallite porosity and the skeletal density variations of HP1 shows that intra-corallite porosity variations are responsible for the growth banding pattern observed in radiographs (Supplementary Figure S1). In contrast to HP1 however, the bulk porosity in W1 is strongly correlated to both the porosity of the corallite (r = 0.90) and the porosity of the theca (r = 0.83), implying that the bulk porosity signal in W1 represents a more balanced ratio between theca and corallite porosity than in HP1. The porosity of the theca was more strongly correlated to skeletal density in W1 than the corallite porosity. This suggests that theca thickening is responsible for the variable skeletal densities observed in W1.

The linear extension rates in HP1 overlap with reported annual extension rates of *S. siderea* sampled from between 6–15 m^26^, supporting our interpretation that the clear porosity cycles in HP1 reflect a seasonally driven growth banding pattern. Upon examining the proportions of pixel classes within each microstructure of HP1, we found a similar correlation between the corallite area and the two-pixel classes (i.e., skeleton and pores). This indicates that as the corallite area fluctuates, both pores and skeleton in each microstructure adjust almost proportionally. Although this proportionality is observed in the pixel classes in the theca region, the stronger correlation between corallite porosity and bulk porosity suggests that seasonal changes in oceanic parameters more dominantly influence processes controlling corallite porosity (i.e., the inter-skeletal/septal spaces).

The slower linear extension rates of W1 also align with records of *S. siderea* growing between 0 and 5 m^26^, yet the porosity cycles are less clear than in HP1. Both pixel classes are the dominant feature in the respective microstructures in W1 (i.e., pixels classified as pores in the corallite and skeleton in the theca). The disproportion in correlation between the classes with the corallite area fluctuation, where pores in the corallite region are more strongly correlated to corallite area, suggests that corallite area drives porosity variations within the corallite. Unlike HP1 however, skeletal pixels in the theca area in W1 are more strongly related to variations in the mean corallite area. Thus, the fluctuation in mean corallite area in W1 is driving both the dominant pixel classes in each respective microstructure. The process(es) influencing the variability in mean corallite area over periods longer than one year in W1 appears to drive both theca thickening and corallite porosity (Fig. 4a-b). As a consequence, this may obscure the shorter-term seasonal patterns in bulk and corallite porosity. In this context, the process(es) which control longer-term corallite area variability in shallower water depths affects carbonate chemistry in both microstructures. This process would thus also affect pH, [CO_3_^2-^], Ω_ar_ upregulation in the theca ^38,45–48^, as well as [HCO_3_^-^] and DIC upregulation in the corallite. Such longer-term variability in corallite area and subsequently bulk porosity only influences W1 which suggests that these parameter(s) is/are water depth dependent (i.e., irradiance^54^ and references there-in).

The formation of seasonal growth bands in *S. siderea* can be related to seasonal thickening in two different ways: 1) seasonal driven variations in the size of the thickening interval in soft tissues, and/or 2) variations in extension rate with a constant tissue thickening interval. The first process refers to cyclic variations in the size and depth of the thickening interval beneath the corallite cup within soft tissues. As mentioned in the introduction, the thickness of soft tissues is reported in literature to be sensitive to seasonality and is expected to be proportionate with the thickening interval beneath the corallites. Assuming a constant calcification and extension rate, a small tissue thickness (and thickening interval) would focus calcification over a small area, thickening a narrow band of skeleton (formation of narrow HDBs). Conversely, a relatively larger CTT would calcify a larger area leading to a bigger growth band with relatively higher skeletal porosities (formation of wide LDBs). A similar effect applies to faster extensions rates which would reduce the time in which skeleton resides in the thickening interval^3^. The combination of both seasonally controlled CTT and extension rates produce relatively wider high porosity bands (LDB) during periods of thicker CTT and faster skeletal extension (i.e., during the summer wet seasons/positive peaks in Fig. 3b) and smaller, less porous skeleton (HDB) in the winter dry season (i.e., negative peaks in porosity Fig. 3b), whereby skeleton spends longer in the thickening interval and calcify a relatively smaller area. Additionally, the process of upregulating [HCO_3_^-^] and DIC in the corallite region is more heavily mediated by organic compounds than in the theca^38,55^. Seasonal variations in oceanic parameters such light and temperature may influence the [HCO_3_^-^] and DIC upregulation in the corallites^42,56,57^, resulting in cyclic porosity cycles in corallite skeleton. Evidence of covariation in calcifying fluid pH and DIC between summer and winter seasons has been reported for *Porites*^58^*.* The clarity of growth corallite cycles in HP1 however suggests that the seasonal driver(s) in tissue thickening and/or corallite carbonate chemistry is/are interannually stable thus producing clear, replicable seasonal bands.

In HP1 there is a similar magnitude decrease in porosity in the thickening interval (pink (ii) to yellow (iii) star in Fig. 6a) to the amplitude of seasonal porosity fluctuations (yellow (iii) to orange (iv) star in Fig. 6a) indicating that the rate of thickening processes must double within a year. Alternatively, variation in the initial thickness of the corallite microstructures (pink star (ii) in Fig. 6a) at the time of the microstructure formation could result in a seasonal porosity signal. This will change the starting porosity point in which is subsequently thickened within the soft tissue thickening interval. The formation and depth of the corallite structures in *S. siderea* were reported to vary in response to pCO_2_, pH, thermal stress^37^, and sedimentation^35,36^. Similar observations were made for another massive coral *Favia fragum* under similar experimental conditions^59^. Specifically, temperature and pCO_2_ are thought to decrease the aspect ratio (i.e., length : width) of the coral’s individual aragonite crystals. This was particularly evident in the septa, causing the septa to narrow. This suggests that the thickness of skeletal components in the corallite, notably the septa, varies during their initial formation and is influenced by environmental parameters. This might also explain the difference in smoothing area, which influenced the corallite boundaries whilst having little effect on the porosity signal. According to this interpretation, seasonality is driven by the initial thickness of the septa during formation, rather than by the thickening of pre-existing skeleton beneath the corallite within the soft tissues.

### Water depth and reef location influences skeletal growth

The differences in clarity of the annual porosity cycles reconstructed for HP1 and W1 is attributed to the 10 m difference in water depth in which the colonies grew. Intra-seasonal environmental parameters are more varied in near-shore reefs (i.e., W1)^60^, which would also contribute to the poor clarity/noise in the seasonal porosity cycles. The growth banding pattern is clearer in HP1, likely due to more stable environmental conditions (as shown by the stationarity in corallite area throughout the sample) and the faster extension rates thus less biological smoothing of the porosity signal^16,23^. For scleractinian corals in general, it is reported that calcification rates, linear extension, and skeletal porosity decrease with decreasing water depth^18,61–63^ with reduced light intensity hypothesised to be the primary factor^26,64,65^. The photosynthetically available radiation (PAR) between 400–700 nm is reported to decrease from 100 % at the surface to 33–36 % at 5 m water depth and to 9–11 % at 20 m^66^. Similarly, PAR decreased from 524.7 W m^-2^ at the surface to 150.4 and 67.9 W m^-2^ at 6 m and 15 m respectively^67^. Downwelling irradiance (i.e., light intensity) is also reported to decrease faster than exponentially in the upper few meters and then exponentially below 10 m^68^. On the north-west coast of Barbados, surface illumination (%) decreased on two reefs from 100 % at the surface to 50 and 40 % at 5 m, and to 30 % and 19 % at 15 m^52^. The strongest variability in light intensity is also most variable between 0.5 and 10 m depths due to surface waves^69,70^. For *S. siderea*, the fastest extension rates have been observed in samples located at a depth between 6–15 m^26^ although this could be related to the observation that colonies in near-shore shallow settings exhibit slower extension rates compared to samples found in forereef settings^71^. Either way, the more dynamic light conditions in shallower water depths in which W1 grew likely influenced the slower extension rates and more variable porosities we observe in the growth banding signal.

Corals growing in higher hydraulic energy environments typical of inshore, shallow reefs have denser skeletons (i.e., lower porosities) to withstand the harsher environment ^72–75^. Under average weather conditions, most wave energy is lost through wave breaking at 2 m water depth (from 1.83 to 1.0 J m^-2^)^76^. During tropical cyclones however, wave energy is reported to increase as high as 5.67 J m^-2^ and begins to dissipate at 5–6 m water depth. The authors who report these values concluded that wave energies have a significant effect on corals growing within 5 m depth, but almost no influence on corals growing deeper than 8 m. W1 displays slower linear extension rates, lower theca porosities, and higher corallite porosities than in HP1, indicating that skeletal thickening is likely focused on reinforcing the theca to strengthen the total skeletal structure in response to higher hydraulic energy at 5 m water depths. A significant difference between the two samples is the porosity variability in the theca, where HP1 displays relatively shorter-term variability with little cyclicity (Fig. 3a) compared to W1 which shows both short- and longer- term variability (Fig. 4a). The two orders of variability within the theca of W1 indicate that thickening is more variable as response to the higher variability in seawater parameters at shallow water depths. The shorter-term variability in the corallite of W1 likely corresponds to the seasonal growth banding pattern. These growth bands are also observed in the porosity cyclicity in the theca, which exhibits longer-term variability consisting of approximately three annual cycles. It is currently unsure what drives this longer-term variability in W1, but it is noticeable absent in HP1. This absence suggests that whatever parameter(s) drives the longer-term variability in W1 is/are specifically magnified in inshore, shallow depth environments (i.e., sedimentation, nutrients, hydrodynamics).

Understanding the differences in growth regimes between the two depths and samples requires an interpretation of whether elevated extension rates are a positive or negative response to environmental stressors. Ceroid corals such as *S. siderea* are thought to invest energy into linear extension to grow quickly and thus any reduction in energy/calcification will be reflected in reduced extension rates^23,77,78^. For example, increased extension rates in response to sedimentation can be interpreted as a positive growth response whereby *S. siderea* derives energy via heterotrophic feeding on suspended particulate matter (SPM; ^35^). Heterotrophic feeding is reported to outweigh the negative energy effects under low light conditions (reduced acquisition of photosynthetically fixed carbon) and elevated SST typical of inshore (turbid) reefs^50^. The more favourable conditions at deeper depths is also supported by the relatively thicker CTT observed in HP1 (Fig. 1), which suggests that HP1 had larger energy stores than W1^50,53^. Higher energy stores with depth is consistent for *S. siderea* growing on the Great Barrier Reef, which report increasing lipid contents in coral soft tissues from 13–30 m^52^. Thus, sedimentation and enhanced heterotrophic feeding may contribute to the increased extension rates at 15 m observed for HP1. Alternatively, fast skeletal extension rates have been reported as a negative response to sedimentation, eutrophication and low light intensity^74,79–81^. This response is termed “stretching modulation”^79^. Fast extension rates during periods of low calcification rates (low energy) result in skeleton with higher porosities. The link between sedimentation and faster extension rates may be explained by fast growing symbiont populations inhibiting homeostatic functions due to high symbiont-host respiration rates^82,83^. This literature would suggest that W1, which extends slower and has lower skeletal porosities, grew in more favourable environmental conditions than HP1 despite the disturbed porosity cyclicity. Thus, depending on the literature perspective and with only two samples from two water depths, it is difficult to state with any certainty the favourability of coral growth at different depths based on solely skeletal extension rates and porosity.

When interpreting coral health based on comparing skeletal porosities from two different colonies, it is important to consider how the degree of smoothing in the image segmentation algorithm influences the reconstructed porosity. Each sample displayed a similar smoothing-related decrease in porosity in the corallite area when smoothed with the larger smoothing area. The smoothing-induced shift in porosity is reversed in the theca area which exhibited a positive increase in porosity when smoothed with the larger area. Both samples, however, showed no significant change in porosity pattern through the depth of the sample. Despite the similar relative difference in smoothing area between the samples, the mean porosity shift between large and small smoothing areas was different (from 56.5 to 55.4 % in HP1 and from 59.52 to 59.4 % in W1; Figs. 3b, 4b respectively). This suggests that smoothing different samples with similar smoothing areas does not have a uniform effect on the yielded porosity which is important when using porosities to compare and infer coral health between different samples. Considering the smoothing area directly influenced the corallite-theca boundary (Fig. 2e-f), the porosity shift highlights the importance of correctly defining the corallite-theca boundary.

## Conclusion

The depth of skeletal thickening is an important aspect to consider when investigating massive scleractinian coral growth strategies and interpreting the growth banding pattern. In this study, we reconstructed porosity within the corallite and theca microstructures along the major growth axis of two *Siderastrea siderea* samples growing at 5 and 15 m water depths. Skeletal porosities were reconstructed using an image segmentation algorithm performed on µCT slices. The reconstructed porosities in the deeper HP1 sample revealed clear, cyclic variations in porosity originating from the corallite microstructures. These porosity cycles are responsible for the growth banding pattern observed on the radiographs and are thought to be driven either by seasonal variations in the width of the thickening interval in soft tissues beneath the corallites or by the thickness of intra-corallite microstructures during their initial formation at the growth surface. The shallower sample (W1) revealed short- and longer-term porosity variability within both the theca and corallite microstructures. Here, corallite growth banding is driven by seasonal variations in corallite area but is masked by other thickening processes. We attribute the differences in the clarity of growth banding between the two samples to the stability of environmental conditions and the resulting energy constancy between the two water depths. We observed that the rate of skeletal thickening differs between the corallite associated skeleton and theca within the first two-thirds of the soft tissues. These findings question the role of the coral’s soft tissues in the apparent timing of porosity banding in massive corals. Future work should try to relate instrumental measurements of depth related parameters such as insolation, wave energy and sedimentation to the thickness of coral soft tissues and, in turn, to the timing of growth banding cycles. Importantly, seasonal growth bands are shown to originate from the corallite region in *S. siderea*, especially in samples living in deeper than 5 m water depths.

## Supplementary Information

Supplementary Information.

## Data Availability

The datasets generated during and/or analysed during the current study are available from the corresponding author upon request.
