# Supplementary material for: Micro-CT analysis reveals porosity driven growth banding in Caribbean coral Siderastrea siderea
Source: Sci Rep. 2025 Feb 19;15:6063. doi: 10.1038/s41598-025-90125-w (PMC11840155; doi:10.1038/s41598-025-90125-w)
Supplement: Supplementary file 1 — Supplementary Information. [file 41598_2025_90125_MOESM1_ESM.pdf]

Micro-CT analysis reveals porosity driven growth banding in Caribbean coral *Siderastrea siderea*

James Vincent*, Tom Sheldrake

james.vincent@unige.ch

Department of Earth Sciences, University of Geneva, Genève, Switzerland

Supplementary Material

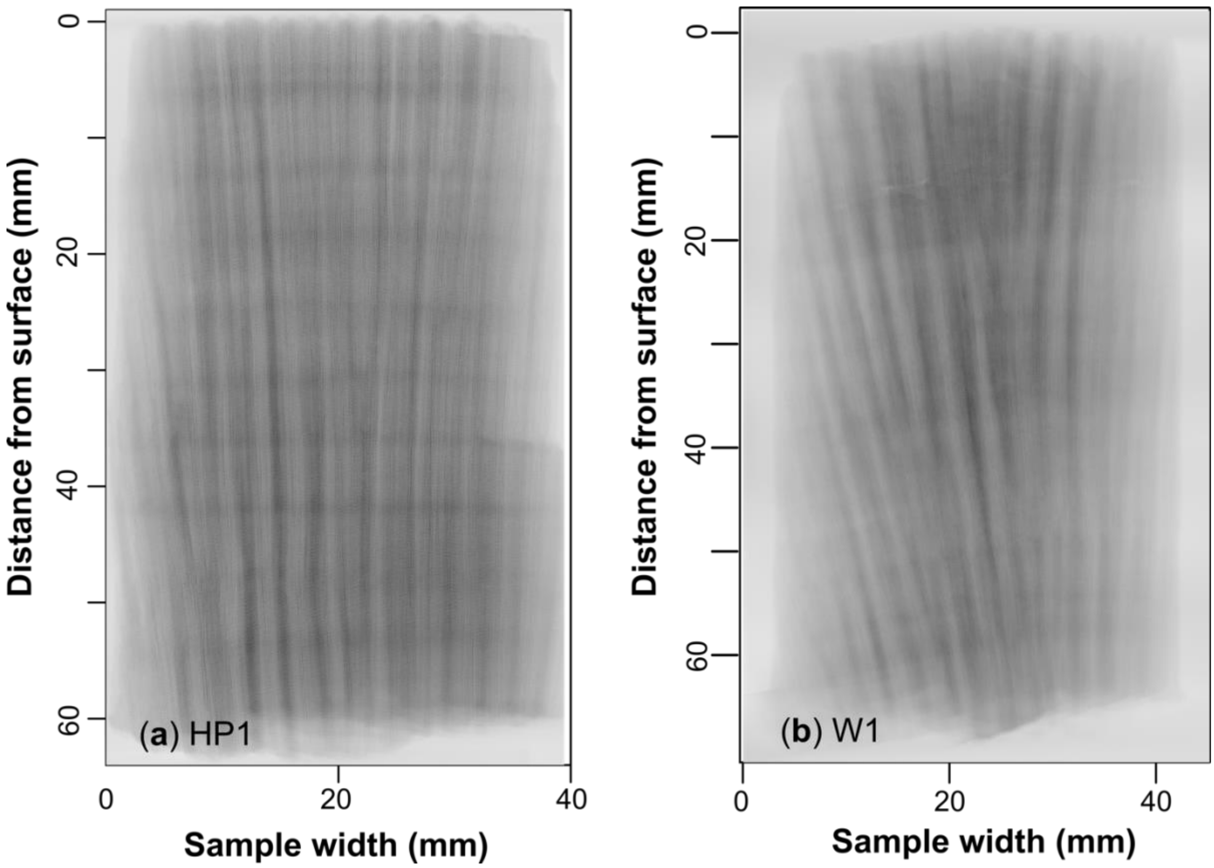

Supplementary Figure 1. Reconstructed radiographs from micro-CT for HP1 (a) and W1 (b). HP1 displays a flat growth surface compared to W1 which shows an uneven growth surface. Additionally, the corallite tracks indicated by the relatively darker vertical lines are straighter in HP1 and angled in W1.

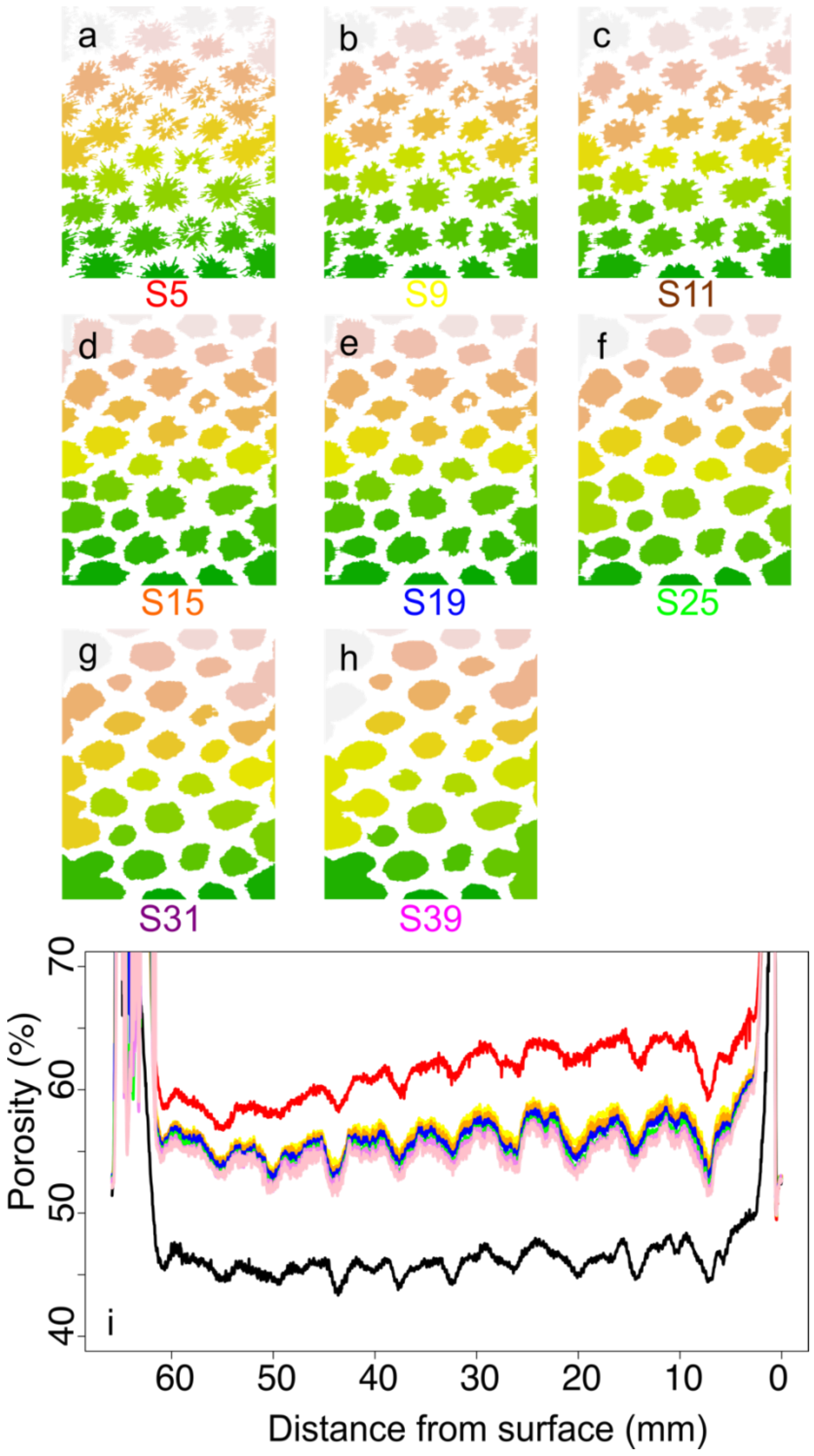

Supplementary Figure 2. HP1 slice #2021 (high density band/low porosity band) smoothed from S=5 to S=39 (panels **a-h**). The corallite porosity reconstructions for the different S-values are displayed in panel **i**. This graph shows that the porosities decrease incrementally towards the complete porosity (i.e., combined theca and corallite) with increasing S. The largest smoothing difference is observed between S5 and S9.

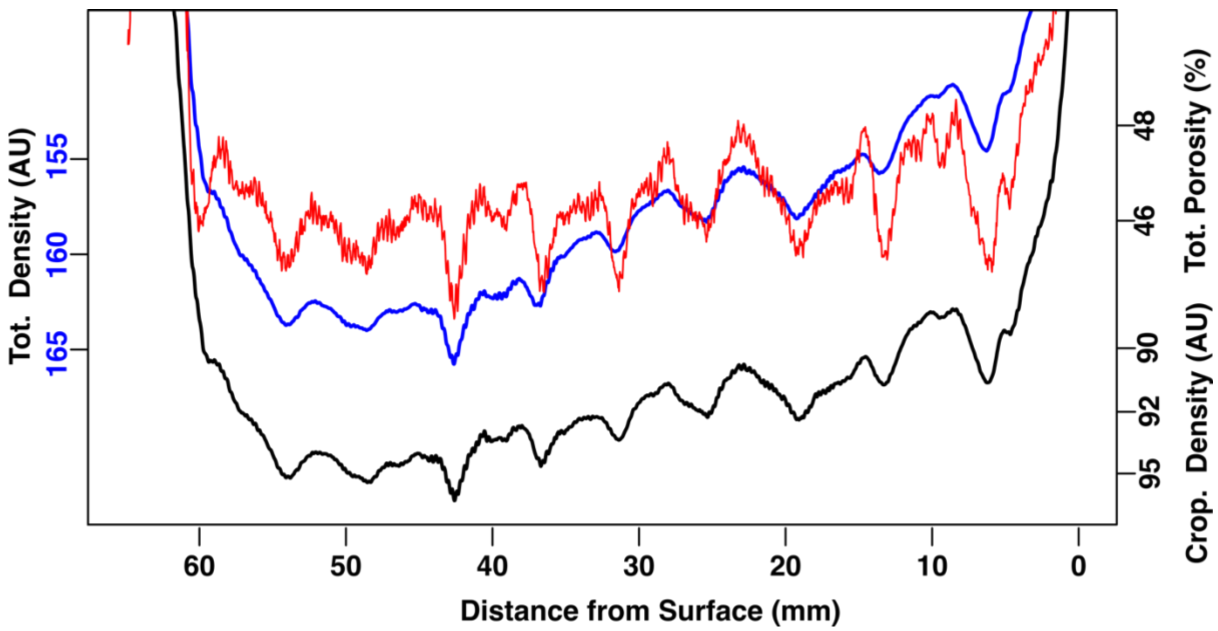

Supplementary Figure 3. Comparing the reconstructed skeletal density of the total sample (blue line - Tot. Density) of HP1 to the skeletal density of the same area used in the pixel segmentation algorithm (black line – Crop. Density). The combined theca and corallite used to reconstruct the complete porosity (red line – Tot. Porosity). Comparing the skeletal density of the total area to the skeletal density of the area used in the pixel segmentation algorithm shows a strong positive correlation (r = 0.99). This correlation shows that the area selected to reconstruct growth banding represents the growth banding patterns of the entire sample. Comparing the same area in the porosity reconstruction (red line) to the density (black line) reconstruction shows a positive correlation (r = -0.81). This shows that the porosity variations are responsible for the growth banding pattern in the radiograph.

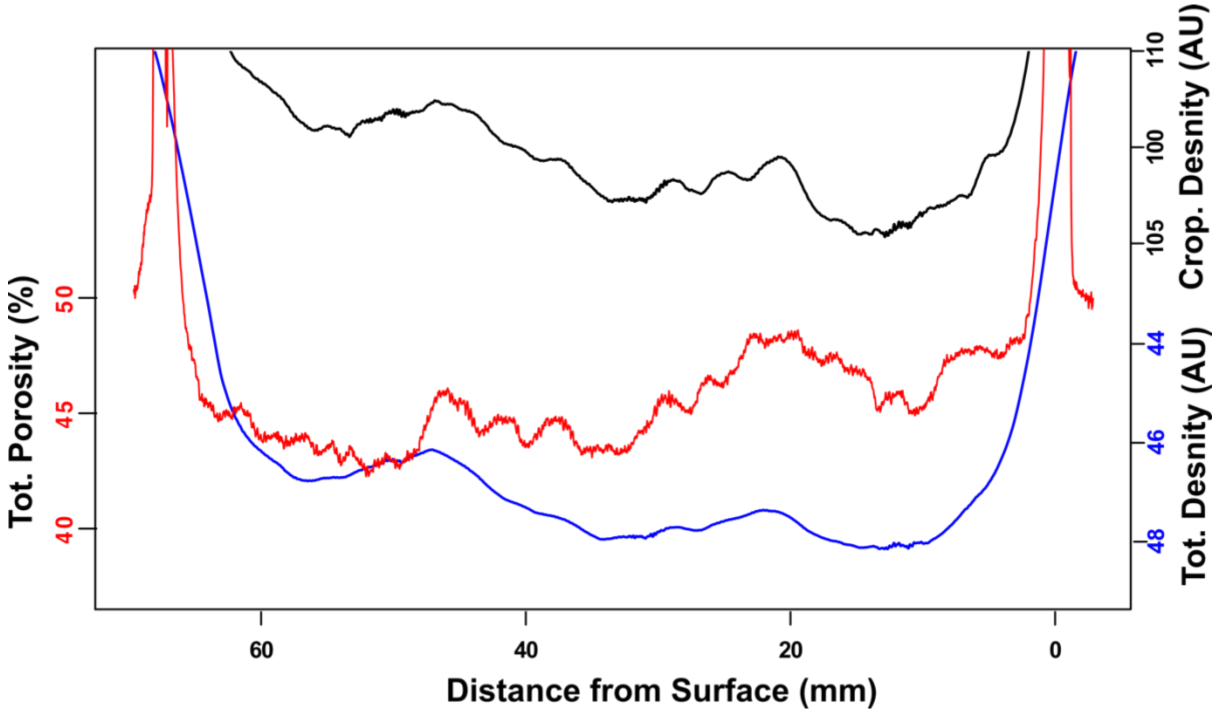

Supplementary Figure 4. Comparing the reconstructed skeletal density of the total sample (blue line - Tot. Density) of W1 to the skeletal density of the same area used in the pixel segmentation algorithm (black line – Crop. Density). The combined theca and corallite used to reconstruct the complete porosity (red line – Tot. Porosity). Comparing the skeletal density of the total area to the skeletal density of the area used in the pixel segmentation algorithm shows a strong positive correlation ($r = 0.99$). This correlation shows that the area selected to reconstruct growth banding represents the growth banding patterns of the entire sample. Comparing the same area in the porosity reconstruction (red line) to the density (black line) reconstruction shows a positive correlation ($r = -0.62$).

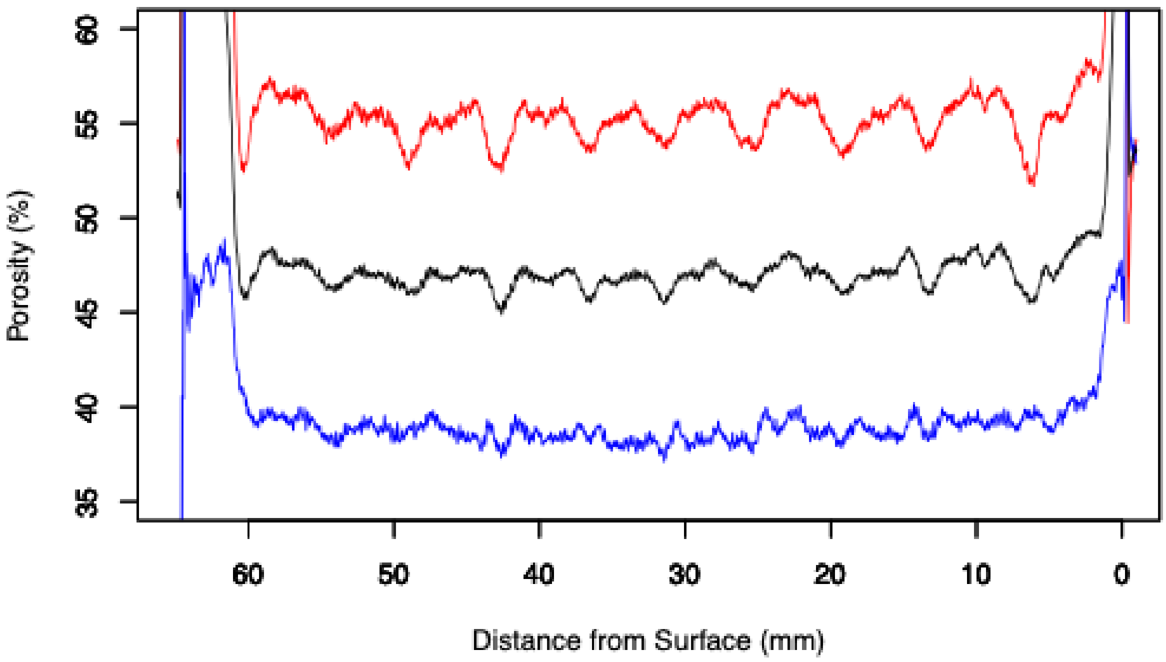

Supplementary Figure 5. Porosity reconstructions on a down-sampled slices with a resolution of 87 μm^2 to show how scan resolutions affect the porosity reconstructions. The corallite, theca and complete porosity (red, blue and black lines respectively) reconstructions from the lower spatial resolution slice (i.e., pre-smoothed) are strongly correlated to the non-smoothed (i.e., resolutions used in the manuscript) for the corallite (r = 0.84), theca (r = 0.85) and total area (r = 0.92). This shows that better resolutions scan would not significantly alter the porosity banding we observe.

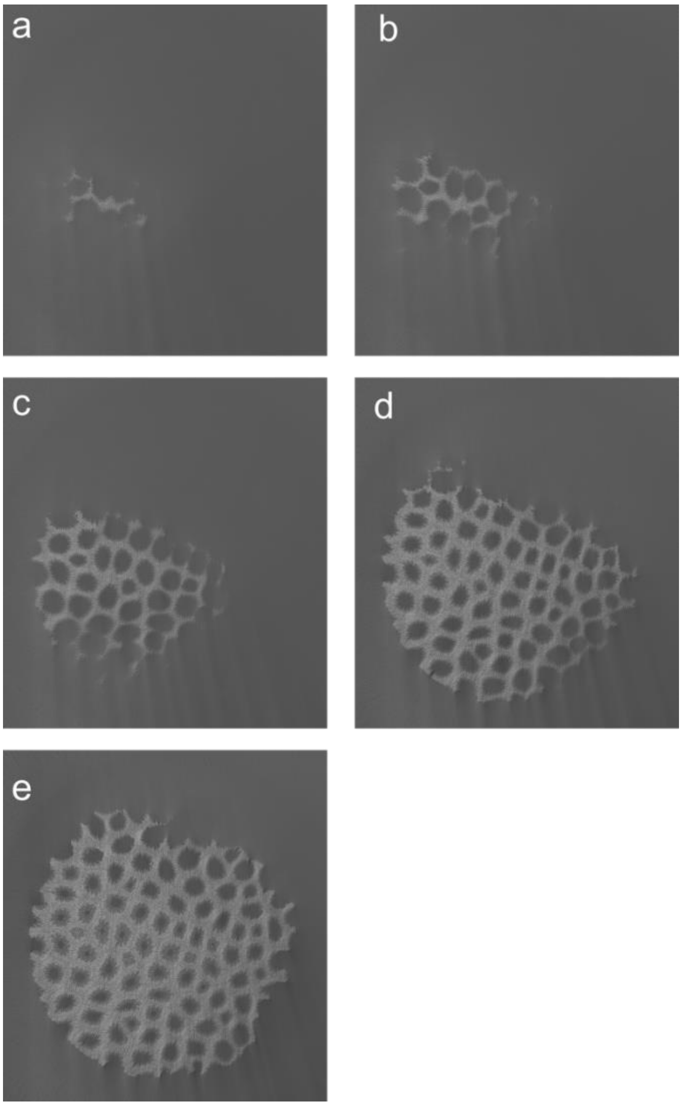

Supplementary Figure 6. W1 slice #61 (**a**), #68 (**b**), #81 (**c**), #107 (**d**) and #133 (**e**) illustrating the growth surface unevenness. These slices show that different parts of the surface of W1 is reconstructed at different depths. Therefore, as corallites begin to form in panel **e**, other corallites have already formed since panel a.
